# *Akkermansia* and its metabolites play key roles in the treatment of campylobacteriosis in mice

**DOI:** 10.3389/fimmu.2022.1061627

**Published:** 2023-01-12

**Authors:** Lai Jiang, Chunchun Yuan, Wenxin Ye, Qixin Huang, Zhuo Chen, Wenzi Wu, Lichun Qian

**Affiliations:** ^1^ Key Laboratory of Animal Nutrition and Feed Science, Ministry of Agriculture and Rural Affairs, College of Animal Sciences, Zhejiang University, Hangzhou, China; ^2^ Hainan Institute of Zhejiang University, Sanya, China

**Keywords:** *Campylobacter jejuni*, fecal microbiota transplantation, *Akkermansia*, butyric acid, deoxycholic acid, PI3K-AKT pathway, MAPK pathway

## Abstract

**Introduction:**

*Campylobacter jejuni* (*C. jejuni*) is a common food-borne bacterial pathogen that can use the host’s innate immune response to induce the development of colitis. There has been some research on the role of normal intestinal flora in *C. jejuni*-induced colitis, but the mechanisms that play a central role in resistance to *C. jejuni* infection have not been explored.

**Methods:**

We treated Campylobacter jejuni-infected mice with fecal microbiota transplantation (FMT), oral butyric acid and deoxycholic acid in a controlled trial and analyzed the possible mechanisms of treatment by a combination of chromatography, immunohistochemistry, fluorescence in situ hybridization, 16s rRNA gene, proteomics and western blot techniques.

**Results:**

We first investigated the therapeutic effect of FMT on *C. jejuni* infection. The results showed that FMT significantly reduced the inflammatory response and blocked the invasion of *C.jejuni* into the colonic tissue. We observed a significant increase in the abundance of Akkermansia in the colon of mice after FMT, as well as a significant increase in the levels of butyric acid and deoxycholic acid. We next demonstrated that oral administration of sodium butyrate or deoxycholic acid had a similar therapeutic effect. Further proteomic analysis showed that *C.jejuni* induced colitis mainly through activation of the PI3K-AKT signaling pathway and MAPK signaling pathway, whereas Akkermansia, the core flora of FMT, and the gut microbial metabolites butyric acid and deoxycholic acid both inhibited these signaling pathways to counteract the infection of *C. jejuni* and alleviate colitis. Finally, we verified the above idea by in vitro cellular assays. In conclusion, FMT is highly effective in the treatment of colitis caused by *C. jejuni*, with which Akkermansia and butyric and deoxycholic acids are closely associated.The present study demonstrates that Akkermansia and butyric and deoxycholic acids are effective in the treatment of colitis caused by *C. jejuni*.

**Discussion:**

This is the first time that Akkermansia has been found to be effective in fighting pathogens, which provides new ideas and insights into the use of FMT to alleviate colitis caused by *C. jejuni* and Akkermansia as a treatment for intestinal sexually transmitted diseases caused by various pathogens.

## Introduction

*Campylobacter jejuni* (*C. jejuni*) is one of the most zoonotic pathogens worldwide in the last decade, with 120,000 human infections reported in the European Union alone in 2020, well ahead of the second highest number, 52,000 cases of *Salmonella* (1). *C. jejuni* is mainly transmitted to humans through contaminated livestock products, including meat, milk and eggs from livestock and poultry. Particularly in poultry, 30.5% and 21.5% of fresh meat samples from broilers and turkeys, respectively, were found to be positive for *Campylobacter* spp. in the EU in 2020, with a few countries reporting infection of up to 62.1% and 58.5% of samples from turkeys and pigs positive for *Campylobacter* spp (1). *C. jejuni* has multiple and potent virulence factors, and healthy people can develop symptoms after infection with only 500 to 800 bacterial units (2). *C. jejuni* colonizes mainly the end of the ileum and colon, invades the colonic epithelium and induces an inflammatory response in the intestinal mucosa (3). The clinical incubation period for C*. jejuni* infection is 2-5 days, followed by mild discomfort, fever, abdominal cramps, myalgia, watery, bloody diarrhea and even inflammatory bowel disease (IBD) (4) and, rarely, severe symptoms such as Bickerstaff’s encephalitis, Miller Fisher’s syndrome, Reiter’s syndrome and Guillian-Barré syndrome (5–7). At the cellular level, *C. jejuni* infection of colonic tissue leads to crypt abscesses, localized ulcers and infiltration of immune cells (especially neutrophils). Although most *C. jejuni* infections are self-limiting and require only symptomatic treatment, a small number of patients with severe symptoms or immunodeficient multimorbidity require antibiotic treatment. There have been some reports that antibiotic treatment can, on the one hand, reduce the diversity of the commensal gut microbiota and, on the other hand, promote the colonization of certain pathogenic bacteria and the development of gastrointestinal infections, with adverse effects on the immune system of vertebrates (8, 9). Therefore, there is a need to find an antibiotic-free way to treat (or prevent) diseases caused by *C. jejuni* in livestock, poultry and humans.

Some studies have suggested possible solutions to this problem: the search for a new type of probiotic that could increase the resistance of the intestine to colonization by pathogenic bacteria (10) or a class of substances with natural antimicrobial activity or that could increase the resistance of the animal organism to pathogens (11). In this context, we are naturally reminded of FMT. FMT is a long-established treatment method that has been documented as early as the fourth century in China for food poisoning and diarrhea. In 2013, FMT was first included in the US clinical guidelines for the treatment of recurrent *Clostridioides difficile* infections (CDI) (12), and it has once again entered the public and mainstream medical landscape, which has led to the realization that gut microbes are vital to animal health, and research into the composition of gut microbes and their relationship to disease has subsequently become a popular direction. 2017 also saw the first recognition of the therapeutic value of FMT for recurrent *Clostridioides difficile* infections in Europe (13). Since then, FMT has been used in clinical studies for the treatment of various diseases, such as obesity (14), diabetes (15), myalgic encephalomyelitis (16), chronic fatigue syndrome (17), constipation (18), irritable bowel syndrome (IBS) (19) and inflammatory bowel disease (IBD) (20). Regarding *C. jejuni*, some wild-type mice have been reported to be protected from infection even after oral administration of highly pathogenic doses of *C. jejuni (* 21), and germ-free mice treated with broad-spectrum antibiotics to eliminate their own intestinal bacteria have acquired resistance to *C. jejuni* infection after transplantation of human feces without apparent symptoms (22).

Based on these results, we hypothesized that FMT could be effective in the treatment of campylobacteriosis due to the presence of probiotics or natural substances with resistance to *C. jejuni* in the animal’s own gut microbes and their metabolite system. We wanted to further investigate the mechanism of FMT in the treatment of *C. jejuni* disease and whether FMT, if used as a treatment, could treat high doses of *C. jejuni* sufficient to cause disease in normal healthy mice. Our study validated the therapeutic efficacy of FMT in campylobacteriosis by combining 16S rRNA gene, targeted metabolomics and proteomics analyses and finally by *in vitro* cellular assays, which revealed that the key to resistance against *C. jejuni* is *Akkermansia* and two important bacterial metabolites, butyric acid and deoxycholic acid.

## Methods

### Chemicals

Butyric acid (≥99.0%, LC) and deoxycholic acid (≥99.0%, LC) were purchased from Sigma–Aldrich.

### Mice

Six-week-old pathogen-free (SPF-grade) male C57BL/6 mice (Hans Biotechnology Co., Ltd., Hangzhou, China), with two mice per cage, were used and housed at a constant temperature of 25°C with a natural light cycle in a standard SPF facility at Zhejiang University. All mouse experiments were carried out in accordance with relevant systems and national regulations to implement guidelines and regulations; the experiments were approved by the Animal Protection and Utilization Committee of Zhejiang University, and all experiments met relevant regulatory standards.

### Bacteria and FMT

The experiment was carried out using *Campylobacter jejuni* (ATCC-33291, ATCC, USA) in broth medium (ATCC Medium 1115, ATCC, USA), rejuvenated in a CO_2_ incubator at 37°C under microaerobic conditions (85% N_2_, 10% CO_2_, 5% O_2_), activated and expanded for 72 h, and diluted to obtain approximately 1.0 x 10^9^ CFU/mL bacterial solution. *Akkermansia* (ATCC-BAA835, ATCC, USA) in brain heart infusion medium (ATCC medium 44, ATCC, USA) was rejuvenated, activated and expanded for 90 h in an anaerobic incubator at 37°C under anaerobic conditions (85% N_2_, 10% CO_2_, 5% H_2_) and diluted to obtain approximately 1.0 x 10^8^ CFU/mL.

For FMT, feces from healthy SPF mice were collected for 14 days as a routine microbiota. Collected fresh feces were immediately suspended in 30% glycerol PBS culture medium, centrifuged at 100 r/min for 2 min, filtered through a 70 μm filter, quantified (OD 600 value of 1.0, predicted to be 10^9^ CFU/mL) and stored at -80°C. Prior to the experiment, all fecal fluids were pooled and homogenized, diluted to 5.0 x 10^9^ CFU/mL and immediately administered to mice by gavage.

### Processing and sample collection

**Figure 1:**
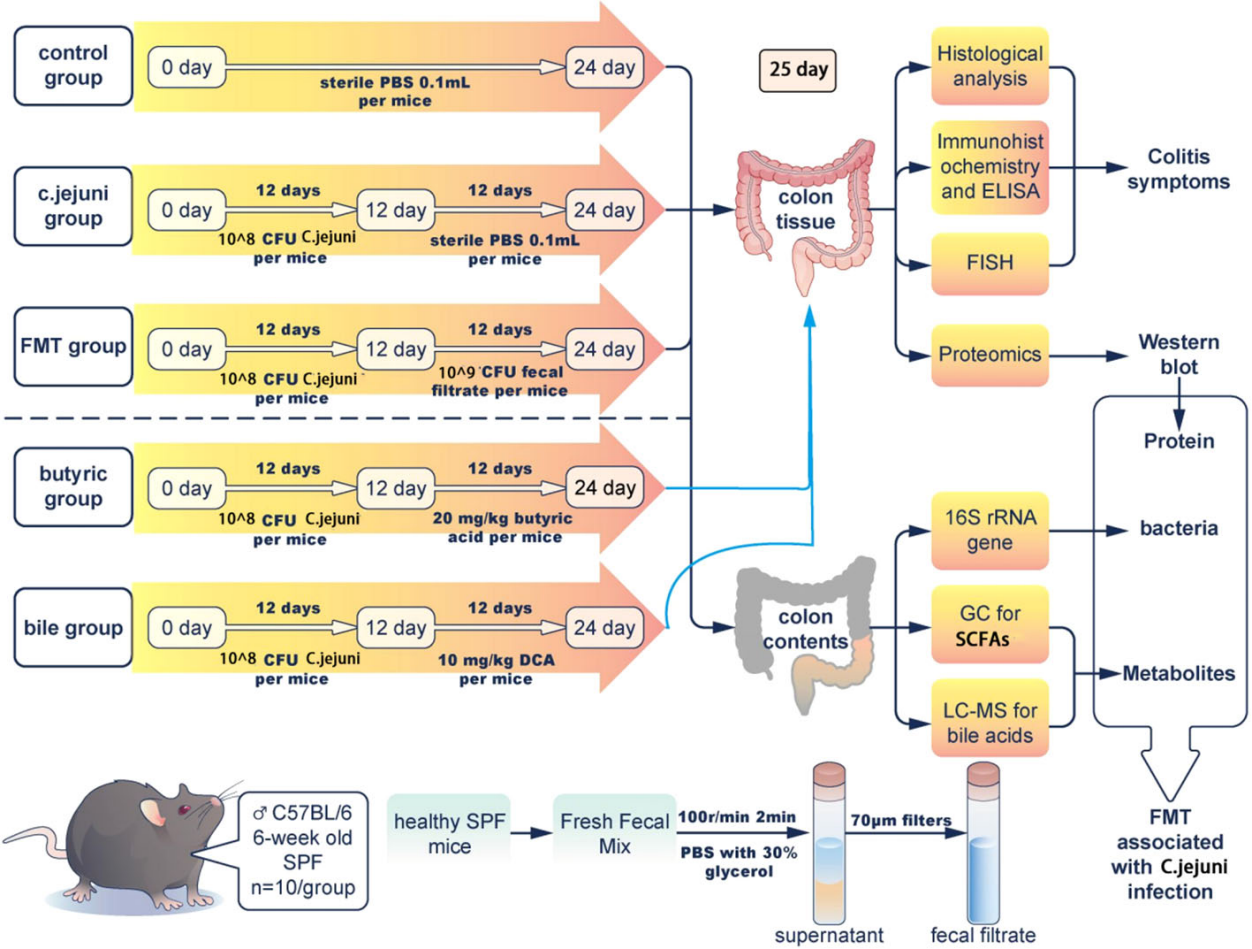
Experiment schematic with n=10 for each group of mice. FMT=fecal microbiota transplantation.

The experimental design is shown in **Figure 1**. After 1 week of acclimatization, the mice were randomly divided into 5 groups of 5 cages each, with 10 mice in each group. The animals were provided ready-to-refill food and drinking water for 24 days, and all preparations(included PBS, *C. jejuni*, FMT preparation, butyric acid and deoxycholic acid) were administered orally. The groups were as follows: (1) Control group, 0.1 mL PBS buffer was administered orally daily for 24 days; (2) *C. jejuni* group, 1.0 x 10^8^ CFU *C. jejuni* per mouse was gavaged daily for 12 days, followed by 0.1 mL PBS buffer daily for 12 days starting on day 13; (3) FMT group, each mouse received 1.0 x 10^8^ CFU *C. jejuni* daily for 12 days, and then 1.0 x 10^9^ CFU fecal bacteria daily for 12 days starting from day 13; (4) Butyric acid group, each mouse received 1.0 x 10^8^ CFU *C. jejuni* per day for 12 days, followed by 20 mg/kg body weight butyrate acid solution per day for 12 days starting from day 13; and (5) Bile acid group, each mouse received 1.0 x 10^8^ CFU *C. jejuni* per day for 12 days, followed by 10 mg/kg body weight deoxycholic acid solution per day for 12 days starting from day 13.

On day 25, mice were anesthetized and sacrificed. Colon tissue, contents of the colon and fresh fecal samples were collected aseptically from all mice and weighed. The colon tissue was divided into the following parts: one part was fixed in 10% formalin for histological analysis; one part was fixed in 2.5% glutaraldehyde for transmission electron microscopy and fluorescence *in situ* hybridization; and the remaining colon tissue was washed three times in sterile PBS and stored at -80°C for analysis.

### Histological analysis

A portion of colon tissue was fixed in 10% formalin and sectioned and stained with hematoxylin and eosin. The tissue was observed under a light microscope (Nikon, Tokyo, Japan), and images were collected for analysis. Images were evaluated using ImageJ software (US National Institutes of Health, Bethesda, MD) to observe the extent of inflammatory infiltration, histopathological changes in the crypt structures, ulceration and loss of crypt, ulceration and the presence of edema. Another portion of colon tissue was fixed with 2.5% glutaraldehyde, sectioned by standard procedures and observed under a transmission electron microscope (Hitachi, Tokyo, Japan), and images were collected for analysis.

### Quantification of indicators of inflammatory factors in the colon

Inflammatory factors, including TNF-α, Cxcl2, IL-6, IL-10 and IL-22, were measured by ELISA. The ELISA kits were purchased from Nanjing Jiancheng Bioengineering Institute (Nanjing, China), and the assays were performed according to the instructions of the kits.

### Immunohistochemistry

Colon tissues were fixed in 10% formalin and heated to boiling in a microwave oven using a repair kit with citric acid antigen repair buffer (pH 6.0). Tissue sections were placed in the repair kit for antigen repair, washed after natural cooling, endogenous peroxidase blocked with 3%BSA(Sigma–Aldrich, USA), incubated with 0.1% primary antibody (MPO,from Abcam,UK), incubated with freshly prepared secondary antibody (HRP-labeled goat anti-rabbit universal secondary antibody,from DAKO, Denmark), washed and then developed with freshly prepared 1%DAB(DAKO, Denmark). Hematoxylin was used to restain the nuclei, which were then dehydrated, sealed, and observed under a light microscope (Nikon, Tokyo, Japan), and images were collected for analysis.

### Fluorescence *in situ* hybridization

Colon tissue samples were fixed in 2.5% glutaraldehyde, and the probe (sequence 5’-UCUAGUAUUGAAGCGUUUUAUGAGUUU -3’) was prepared in 1:100 buffer dilution to make a hybridization solution, which was added dropwise for specific hybridization. This step was followed by dropwise addition of biotinylated mouse anti-digoxin, DAPI restaining of cell nuclei and finally sealing of slices with anti-fluorescence quenching sealer. Sections were observed under a Nikon inverted fluorescence microscope, and images were collected.

### DNA extraction, 16S rRNA gene sequencing and data processing

Total DNA extraction from the microbial community was performed according to the instructions of the E.Z.N.A.^®^ soil DNA kit (Omega Bio-Tek, Norcross, GA, U.S.), PCR amplification of the variable regions of the 16S rRNA gene V3-V4 (338F and 806R) was performed, followed by stable extension at 72°C for 10 min and storage at 4°C. (PCR instrument: ABI GeneAmp^®^ Model 9700). The recovered products were purified using the AxyPrep DNA Gel Extraction Kit (Axygen Biosciences, Union City, CA, USA), detected by 2% agarose gel electrophoresis and quantified using a Quantus™ Fluorometer (Promega, USA). The recovered products were quantified by a Quantus™ Fluorometer (Promega, USA). Library construction was carried out using the NEXTflexTM Rapid DNA-Seq Kit (Bioo Scientific, USA). Sequencing was performed using Illumina’s MiSeq PE300/NovaSeq PE250 platform (Major Bio, Shanghai China).

QC of raw sequences was conducted using fastp (https://github.com/OpenGene/fastp, version 0.20.0) software and FLASH (http://www.cbcb.umd.edu/software/flash, version 1.2.7), which was used to perform splicing: bases were filtered with quality values below 20 in the tail of the reads, the reads with quality control values below 50 bp were filtered, and the reads containing N bases were removed; according to the overlap relationship between PE reads, pairs of reads were spliced (merged) into one sequence with a minimum overlap length of 10 bp; the maximum error allowed in the overlap region of the spliced sequence was also the maximum mismatch ratio allowed in the overlap region, 0.2, and the nonconforming sequences were screened; the samples were differentiated according to the barcode and primers at the beginning and end of the sequence, and the sequence orientation was adjusted, with the number of mismatches allowed for the barcode being 0 and the maximum number of primer mismatches being 2.

Sequences were OTU clustered, and chimeras were removed based on 97% similarity using UPARSE software (http://drive5.com/uparse, version 7.1). Each sequence was annotated for species classification using the RDP classifier (http://rdp.cme.msu.edu, version 2.2) compared to the Silva 16S rRNA database (version 138), and a comparison threshold of 70% was set.

### GC for SFCAs

SCFAs (including acetic, propionic, n-butyric, isobutyric, valeric and isovaleric acids) were quantified in faecal samples using a gas chromatograph. Briefly, 50 mg of fresh faecal sample was weighed, 50 mL of ultrapure water was added, vortexed and mixed, and then centrifuged at 3 000×g for 10 minutes. The supernatant was transferred and treated with 25% metaphosphoric acid (Sigma-Aldrich, USA) at v:v=9:1 for 3h, then filtered through a sterile 0.22 mm membrane and stored in a 2 mL screw-cap vial before analysis on a gas chromatograph (G1540N, HP, California, USA) on a DB-FFAP (HP The column was a DB-FFAP (HP, California, USA), 30 m× 250 μm, 0.25 μm; the carrier gas was high purity nitrogen (99.999%) at a flow rate of 0.8 mL/min; the auxiliary gas was high purity hydrogen (99.999%); the detector FID temperature was 280°C, the inlet temperature was 250°C, the splitting ratio was 50:1, and the injection volume was 1 μL; the programmed ramp-up: the initial temperature was 60°C, and the ramp-up was at a rate of 20°C/min to 220°C. The standards of SCFAs (including acetic acid, propionic acid, n-butyric acid, isobutyric acid, valeric acid and isovaleric acid from Sigma-Aldrich, USA) were prepared in ultrapure water at 25, 50, 100, 250, 500 and 1000 μg/mL (isobutyric acid, valeric acid and isovaleric acid). (5, 10, 25, 50, 100, 250, 250 μg/mL for isobutyric, pentanoic and isovaleric acids)

### LC-MS for bile acids

Quantification of bile acids in faecal samples using UHPLC-MS. Briefly, a 50 mg fresh faecal sample was weighed, dissolved in methanol solution, homogenised and then centrifuged at 3 000×g for 10 minutes. The supernatant was filtered through a sterile 0.22 mm membrane and stored in a 2 ml screw-cap vial.The bile acids were then analysed using ultra performance liquid chromatography-mass spectrometry (LCMS2020, Shimadzu, Kyoto, Japan) and SIMCA software (V16.0.2, Sartorius Stedim Data Analytics AB, Umea, Sweden).

The chromatographic conditions were: Shim-packUCX-RP column (2.1 mm × 150 mm, 3 μm) (Shimadzu, Kyoto, Japan) at 40°C. Mobile phase A was water (containing acetic acid, pH 4.3); mobile phase B was 90% methanol in water (containing acetic acid, pH=4.3). The gradient elution program was 0 min: 80% B; 12 min, 89% B; 14 min, 95% B; 15 min, 80% B; 20 min, 80% B.

The mass spectrometry conditions were: negative ion scanning mode was used. The atomisation gas pressure was 0 33 m Pa (48 psi), the collision gas pressure was 0.7 m Pa, the sheath gas temperature was 350°C, the sheath gas flow rate was 11 L/min, the dryer temperature was 325°C, the dry gas flow rate was 8 L/min, and the detection was in multiple reaction monitoring mode.

Each standard (containing 38 bile acids, available in original data-LCMS data for bile acids, from Sigma-Aldrich, USA) was dissolved in methanol and solutions of 10, 25, 50, 100 and 250 μg/mL were prepared at different ratios.

### Proteomics and data analysis

Total protein was obtained from colon tissue samples using T-PER Tissue Protein Extraction Reagent (Thermo Fisher 78510) and quantified using a Pierce BCA Kit (Thermo Fisher 23225). A 100 μg sample of protein was taken, and the volume was replenished to 90 µl with lysate. TCEP reductant (10 mmol/L) was added at a final concentration, and the reaction was carried out at 37°C for 60 min. Then, 40 mmol/L iodoacetamide was added at a final concentration, and the reaction was carried out at room temperature and protected from light for 40 min. Precooled acetone (acetone:sample volume ratio = 6:1) was added to each tube and precipitated at -20°C for 4 h. The precipitate was centrifuged at 10,000×g. The precipitate was centrifuged at 10,000×g for 20 min. The samples were fully dissolved with 50 mmol/L TEAB, and trypsin was added at a mass ratio of 1:50 (enzyme:protein) and digested overnight at 37°C.

Add one tube of TMT reagent (TMT10-126 labeled, 127N labeled A2, 127C labeled A3, 128N labeled B1) per 100 µg of peptide. The tubes were incubated at room temperature for 2 hours, hydroxylamine was added, and the tubes were allowed to react at room temperature for 15 min. Equal amounts of labeled product were mixed in one tube and evacuated dry in a vacuum concentrator.

The peptide samples were resolubilized with UPLC loading buffer and subjected to high pH liquid phase separation using a reversed-phase C18 column. 2% acetonitrile (ammonia to pH 10) for phase A and 80% acetonitrile (ammonia to pH 10) for phase B. 0~2 min, 100% A; 2~17 min, 0~3.8% B; 17~35 min, 3.8~24% B; and 35~38 min, 24~30% B. UV detection was conducted at 214 nm, the volume flow rate was 200 μL/min, and the elution time was 66 min. Twenty fractions were collected according to peak shape and time, combined into 10 fractions and concentrated by vacuum centrifugation.

The second dimension was analyzed by sodium upgrading liquid chromatography tandem mass spectrometry (Easy-nLC 1200 combined with a Q Exactive mass spectrometer). The peptides were solubilized with mass spectrometry loading buffer and sampled and separated by a C18 column (75 μm × 25 cm, Thermo, USA) for 120 min at a volume flow rate of 300 μL/min. The EASY-nLC mobile phases were 2% acetonitrile (plus 0.1% formic acid) as mobile phase A and 80% acetonitrile (plus 0.1% formic acid) as mobile phase B, with the following gradient: 0~1 min, 0~5% B; 1 ~63min, 5%~23% B; 63~88 min, 23%~48% B; 88~89min, 48%~100% B; 89~95 min, 100% B. MS and MS/MS acquisition were both conducted, with mass spectral resolutions of 70 K and 35 K, respectively. Secondary fragmentation was performed with a dynamic exclusion time of 18 s.

The raw files from the mass spectrometry downstream were analyzed by Proteome Discoverer TM Software 2.2 (Thermo, Waltham, Mass, USA). The search species was mouse, and the database was https://www.uniprot.org. The false discovery rate (FDR) for peptide identification during the search was set to FDR ≤ 1%. Proteins contained at least one specific peptide. P values for significant differences between samples were calculated using the t test function in the R language, and the fold change (FC) was calculated for differences between groups. Significantly differentially expressed proteins were selected as follows: P < 0.05 and FC > 1.2 for upregulated proteins and P < 0.05 and FC < 0.83 for downregulated proteins.

### Cell proliferation-toxicity test

Three different invasion assay procedures (competition, rejection and replacement) and two drug treatment procedures (butyric acid and DCA) were conducted. Briefly, mouse primary colonic epithelial cells (MUCS) were seeded at 1x10^5^ cells per well in 24-well tissue culture plates. Cell monolayers were washed with PBS and fresh RPMI 1640 medium supplemented with 10% FBS (no antibiotics). MUCS grown in 24-well tissue culture plates were infected with 1x10^8^ CFU C*. jejuni* and *Akkermansia* spp. and incubated for 12 hours as a control. For the competition test, 1x10^8^ CFU *C. jejuni* and *Akkermansia* were added together to the cell cultures and incubated at 37°C for 12 hours; for the substitution test, cells were incubated with 1x10^8^ CFU *C. jejuni* for 6 hours at 37°C followed by 1x10^8^ CFU *Akkermansia* for 6 hours; for the rejection test, cells were added at 37°C with 1x10^8^ CFU *Akkermansia* for 6 hours, followed by 1x10^8^ CFU *C. jejuni* for 6 hours; for the butyric acid treatment test, cells were incubated with 1x10^8^ CFU *C. jejuni* at 37°C for 6 hours, followed by 20 mg/mL butyric acid for 6 hours; for the deoxycholic acid treatment test, cells were incubated with 1x10^8^ CFU *C. jejuni* at 37°C for 6 hours followed by the addition of 10 mg/mL deoxycholic acid for 6 hours. Each group of cells was then assayed for cell proliferation toxicity using the CCK8 assay. The CCK8 kit (Sigma–Aldrich, USA) was used according to the instructions provided by the manufacturer.

### Western blot

Colon tissue was washed 3 times with cold PBS to remove blood, cut into small pieces, added to 10 times the volume of tissue lysate, homogenized on ice and then immunoblotted by standard procedures using mouse anti-RTK, anti-ITGB, anti-PI3K, anti-Akt, anti-ERK1/2, anti-JNK, anti-p38 antibodies and mouse anti-β-actin antibody (Abbkine).

### Statistical analysis

All data are expressed as the mean (or mean as a percentage) and were analyzed using the GraphPad Prism 8.0 program (GraphPad Software, San Diego, Canada). One-way ANOVA and Tukey’s multiple comparison tests were used to compare data between two or more groups. Adjusted *P* values below 0.05 were considered statistically significant.

## Results

### FMT alleviates *Campylobacter jejuni*-induced colitis

**Figure 2:**
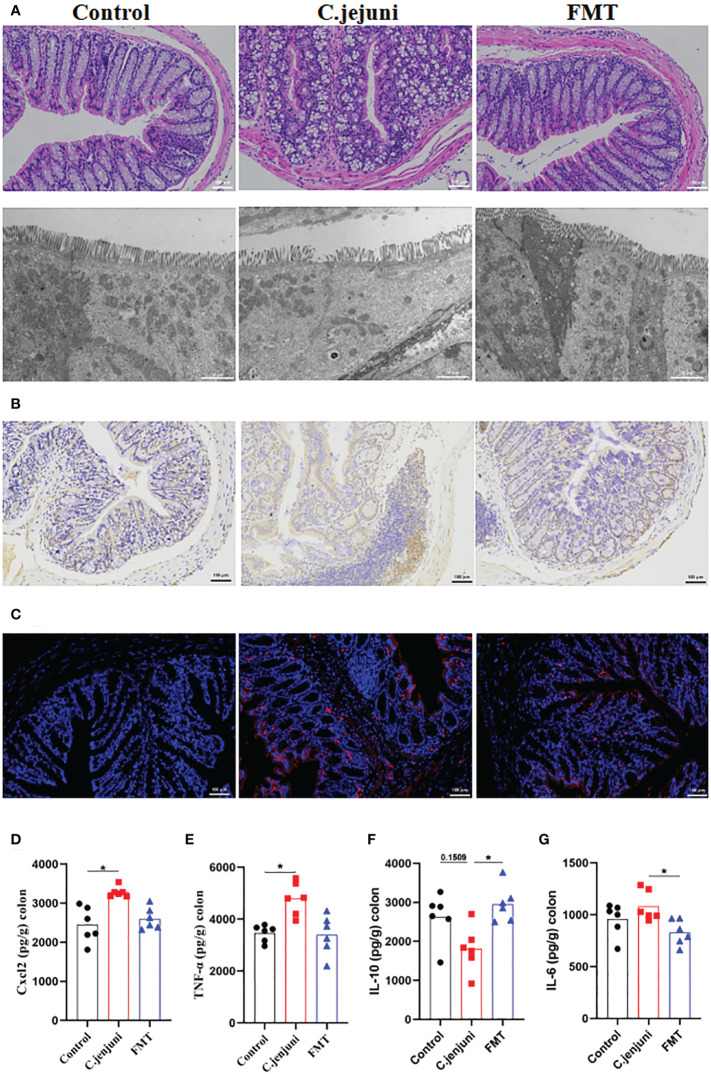
FMT alleviates *C. jejuni*-induced colitis. **(A)** Representative image of colonic tissue using H&E staining at a 200 μm field of view and transmission electron microscopy at a 2 μm field of view. **(B)** Representative image of colon tissue paraffin section MPO immunohistochemical staining (positive for brown) at 100 μm field of view. **(C)** Representative fluorescent *in situ* hybridization of selected *C. jejuni* DNA fragments from colonic tissue after representative fluorescent images at 100 μm field of view. Concentrations of Cxcl2 **(D)**, TNF-α **(E)**, IL-10 **(F)** and IL-6 **(G)** in colon tissues from each group (n=6). Statistical significance was determined using one-way ANOVA, followed by Tukey’s test. *P ≤ 0.05.

In our experiments we observed weight loss, reduced food intake, loose stools and depression in mice persistently infected with Campylobacter jejuni. According to the histological pictures, the colonic tissue of the mice in the Campylobacter jejuni group was damaged, the cells were disorganised, the number and depth of crypt foci were reduced, there was an abnormal increase in the number of intestinal glands, mucus exudate was observed in the intestinal lumen and the microvilli structure was damaged by transmission electron microscopy. This is a more typical symptom of catarrhal inflammation, an exudative inflammation of the mucosal tissues, with clinical manifestations close to those we observed in mice. In contrast, the colonic tissue of the control and faecal transplant groups was more intact overall, with clearly visible crypt foci, no abnormal proliferation of intestinal glands and more neatly arranged microvilli on transmission electron microscopy (**Figure 2A**). As neutrophils play a key role in *C. jejuni*-induced colitis, we used immunohistochemistry to assess the status of MPO-positive neutrophil infiltration in colonic tissue. The results showed that FMT significantly reduced the migration of MPO-positive neutrophils into the colonic tissue (**Figure 2B**). FISH results showed that *C. jejuni* invaded the colonic tissue of infected mice in large numbers, while FMT improved this situation, with only a small proportion of *C. jejuni* remaining in the colonic tissue (**Figure 2C**). Compared to uninfected mice, *C. jejuni* infection significantly increased the levels of Cxcl2 (**Figure 2D**) and TNF-α (**Figure 2E**) in colonic tissue, and FMT significantly increased the levels of IL-10 (**Figure 2F**) and decreased the levels of IL-6 (**Figure 2G**) in colonic tissue.

### FMT improves the microbial composition of the gut and promotes the production of butyric and deoxycholic acids

**Figure 3:**
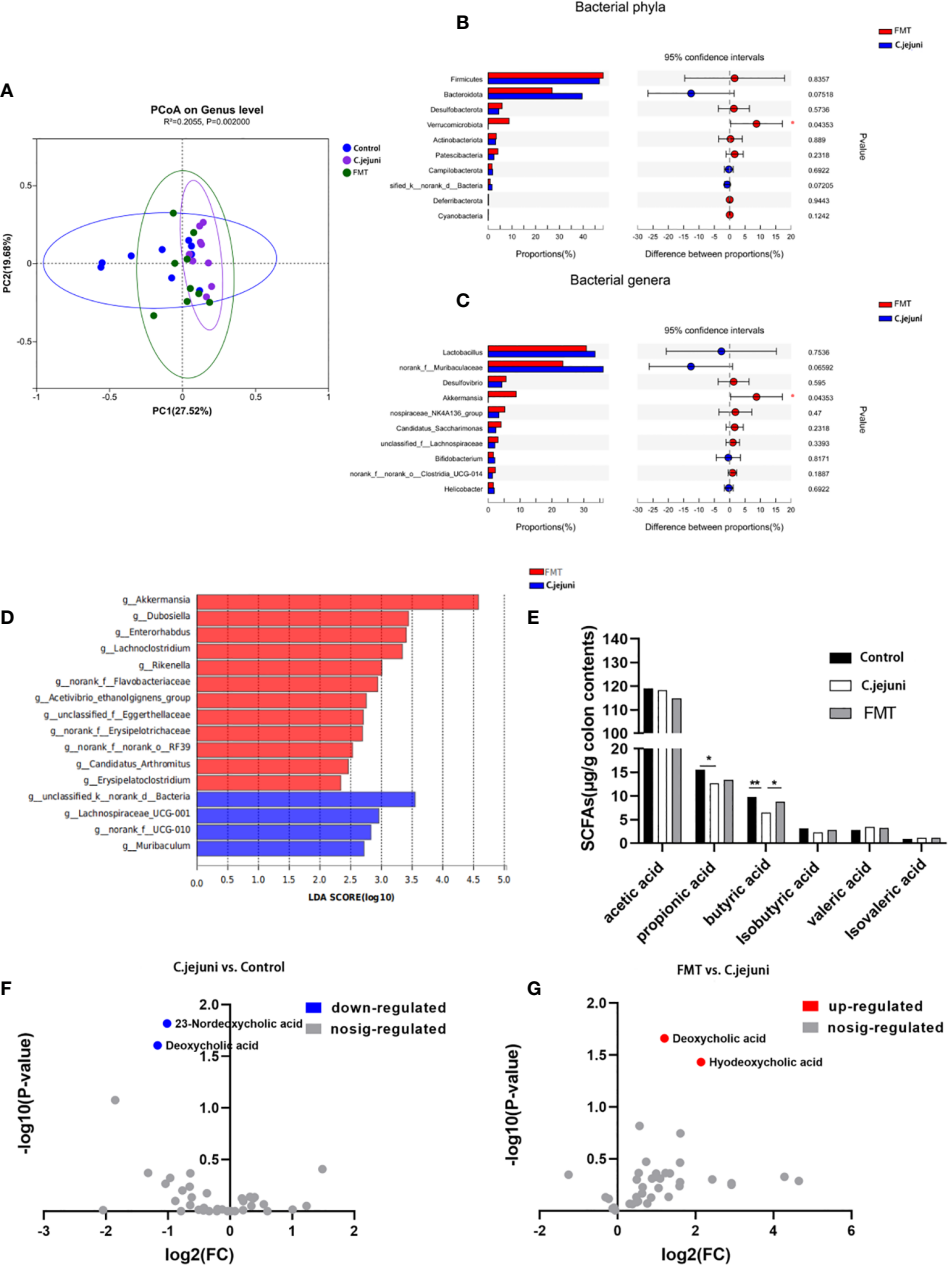
FMT regulates the composition of the intestinal microbiota and the production of butyric acid and deoxycholic acid. **(A)** PCoA plots were assessed by PERMANOVA between the three groups. The relative abundance of colon contents at the bacterial phylum level **(B)** and genus level **(C)** occurred in 99.5% of the communities. Analysis of differences in microbial classification between the FMT group and the *C. jejuni* group **(D)** was shown using LEfSe analysis (LDA combined with effect size measurements). Concentrations of each SFA in colon contents **(E)**, n = 6 for each group. Changes in bile acids in colon contents of control and *C. jejuni* groups **(F)**, Changes in bile acids in colon contents of FMT and *C. jejuni* groups **(G)**, n = 3 for each group. Statistical significance was determined using one-way ANOVA, followed by Tukey’s test. *P ≤ 0.05, **P ≤ 0.01.

We further explored the effect of FMT treatment on the intestinal microbial composition of *C. jejuni*-infected mice by 16S rRNA gene sequencing, obtaining a total of 9659854 sequences with a total length of 4055337852 bp. The Shannon index (**Figure S1A**), Chao index (**Figure S1B**) and Sobs index (**Figure S1C**) of samples from different groups were broadly similar, with no significant differences in alpha diversity between groups. Principal coordinate analysis (PCoA) based on Bray-Curtis distances showed a significant separation in microbial community structure between the three groups of mice (R^2 =^ 0.2055, *P*=0.002, **Figure 3A**), with a highly significant separation in the C. jejuni group compared to the Control group (R^2 =^ 0.2275, *P*=0.001, **Figure S1D**), and in the FMT group (R^2 =^ 0.1525, *P*=0.03, **Figure S1E**) and the microbial community structure of the FMT group was not significantly separated compared to the Control group (R^2 =^ 0.1059, *P*=0.127, **Figure S1F**), thus indicating that the FMT preparation worked to regulate the structure of the intestinal flora that had been altered by the influence of *C. jejuni* and to restore it to normal.

At the phylum level, *Firmicutes*, *Bacteroidota* and *Verrucomicrobiota* were the main phylum in healthy mice, and infection with C. jejuni resulted in a highly significant increase in the relative abundance of Bacteroidota (*P*<0.01), The relative abundance of *Firmicutes* and *Desulfobacterota* increased but not significantly (*P*>0.05), and the relative abundance of *Verrucomicrobiota* decreased significantly (*P*<0.05)(**Figure S1G**). At the genus level, *Muribaculaceae*, *Lactobacillus* and *Akkermansia* were the dominant genera in healthy mice. After infection with *C. jejuni*, the relative abundances of *Muribaculaceae* (*P*<0.01) and *Lactobacillus* (*P*<0.1) increased, and the relative abundance of *Akkermansia* (*P*<0.05) decreased (**Figure S1H**). Using LEfSe analysis, we identified bacterial groups with significantly different abundances in the colons of *C. jejuni*-infected and healthy mice. *Akkermansia*, *Peptococcus* and *Turicibacter* abundances were enriched in the healthy mouse gut, and three bacterial genera were enriched in the *C. jejuni* group (**Figure S1I**).

For the FMT preparation used in the trial, we found by 16S rRNA gene analysis that there was no significant difference between its bacterial composition and the main intestinal flora of the Control group at both the phylum level and the genus level (*P*>0.05, **Figures S1J, K**). Considering that the FMT preparation takes some time even when ready to use and is removed from the anaerobic environment of the gut, which can cause some loss of anaerobic bacteria in the composition, it is acceptable that the abundance of anaerobic bacteria represented by *Akkermansia* (genus level) and *Verrucomicrobiota* (genus level) belonging to the FMT preparation is reduced but not significant.

After treatment with FMT, the relative abundances of *Firmicutes* (*P*>0.05)*, Desulfobacterota* (*P*>0.05)*, and Verrucomicrobiota* (*P*<0.05) *increased*, and the relative abundance of *Bacteroidota* (*P*>0.05) *decreased at* the phylum level (**Figure 3B**); at the genus level, the relative abundances of *Desulfovibrio* (*P*>0.05) and *Akkermansia* (*P*<0.05) increased, and the relative abundances of *Lactobacillus* (*P*>0.05) and *norank_f_Muribaculaceae* (*P*>0.05) decreased (**Figure 3C**). Using LEfSe analysis, we also identified groups of bacteria that differed significantly in abundance between *C. jejuni* infection and FMT treatment. Twelve bacterial genera, including *Akkermansia*, were enriched after FMT treatment, and four bacterial genera were enriched in the *C. jejuni group* (**Figure 3D**). Combining these results, we can see that *Akkermansia* is the core bacterial genus for FMT treatment, and we believe it plays a key role in the colonization by *C. jejuni* and in the treatment.

We wanted to further investigate whether there was also an effect on bacterial metabolites such as SCFAs and bile acids in the attack and FMT treatment. We first analyzed the content of SCFAs in the colonic contents of mice using gas chromatography and showed that the concentration of propionic acid in the colon was significantly reduced and the concentration of butyric acid was highly significantly reduced after infection with *C. jejuni*, whereas the concentration of butyric acid was significantly increased after FMT (**Figure 3E**). We subsequently analyzed the composition of bile acids in the colonic contents of mice using high-performance liquid chromatography–mass spectrometry and showed that the concentrations of deoxycholic acid and 23-nordeoxycholic acid in the colon were significantly reduced after infection with *C. jejuni* (**Figure 3F**), whereas the concentrations of deoxycholic acid and hyodeoxycholic acid were significantly increased after FMT (**Figure 3G**). Butyric acid and deoxycholic acid were two substances that decreased following *C. jejuni* infection and increased following FMT, thus demonstrating the important role of both in the mechanism of FMT for *C. jejuni* infection.

Based on the above results, we can see that the average proportion of *Akkermansia* in the Control group was about 25% (**Figure S1H**), while in the C. jejuni group the proportion decreased sharply to less than 1% (**Figure S1H**) and increased to about 10% in the FMT group (**Figure 3C**). At the genus level, we only observed this variation in *Akkermansia*, and *Akkermansia* was also the only genus to show a significant difference in the top 10 mean sums in the comparison (both the Control group vs. the C.jejuni group, and the C.jejuni group vs. the FMT group). We therefore consider *Akkermansia* to be one of the core genera for microbial community variation. The changes in butyric and DCA concentrations in the colon in the trial were consistent with changes in the abundance of *Akkermansia*, from which we suggest that the decrease in *Akkermansia* abundance and the decrease in butyric and DCA concentrations may be closely related to the infestation of *C.jejuni*, and that the key to FMT treatment is to increase the levels of *Akkermansia*, butyric and DCA in the colon.

### Therapeutic effect of butyric acid on *Campylobacter jejuni*-induced colitis

**Figure 4:**
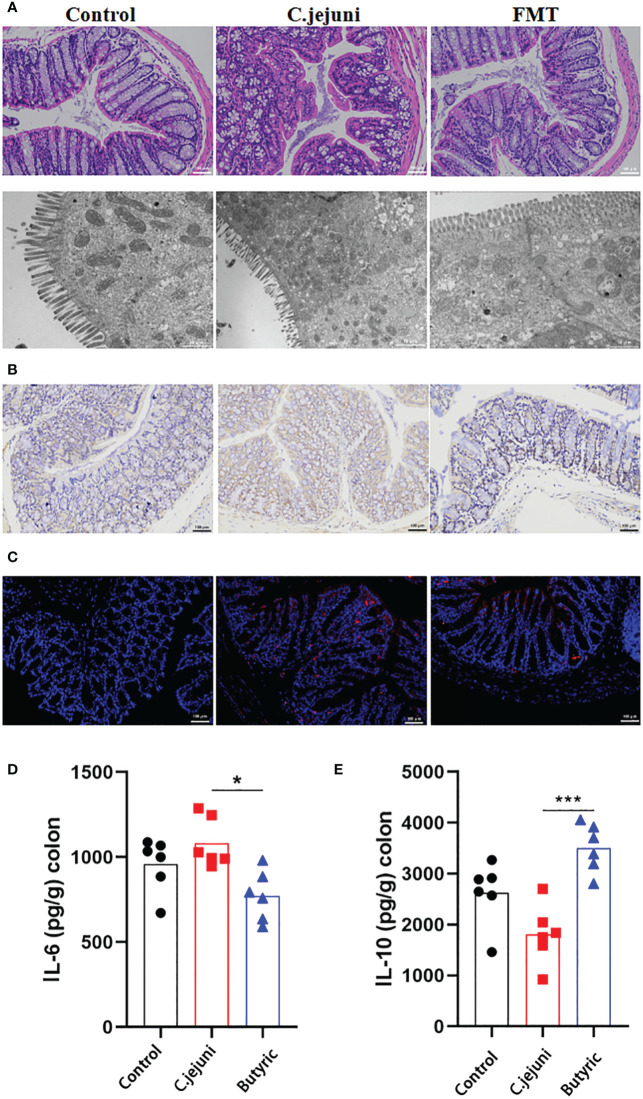
Oral butyric acid relieves *C. jejuni*-induced colitis. **(A)** Representative image of colonic tissue using H&E staining at a 200 μm field of view and transmission electron microscopy at a 2 μm field of view. **(B)** Representative image of colon tissue paraffin section MPO immunohistochemical staining (positive for brown) at 100 μm field of view. **(C)** Representative fluorescent *in situ* hybridization of selected *C. jejuni* DNA fragments from colonic tissue after representative fluorescent images at 100 μm field of view. Concentrations of IL-6 **(D)** and IL-10 **(E)** in colon tissues from each group (n=6). Statistical significance was determined using one-way ANOVA, followed by Tukey’s test. *P ≤ 0.05, ***P ≤ 0.001.

The short-chain fatty acid group has previously been reported to be useful in the treatment of *C. jejuni* infection. We further investigated whether butyric acid also has a therapeutic effect on *C. jejuni* infection. We administered freshly prepared sterile butyric acid solution (20 mg/kg body weight) by gavage to SPF mice infected with *C. jejuni* for 12 consecutive days and observed the therapeutic effect. The results showed that butyric acid treatment alleviated the symptoms of colitis (**Figure 4A**), reduced the migration of MPO-positive neutrophils into the colonic tissue (**Figure 4B**), and similarly reduced the infection of *C. jejuni* (**Figure 4C**). Butyric acid also significantly reduced IL-6 levels (**Figure 4D**) and significantly increased IL-10 levels (**Figure 4E**).

### Therapeutic effect of deoxycholic acid on *Campylobacter jejuni*-induced colitis

**Figure 5:**
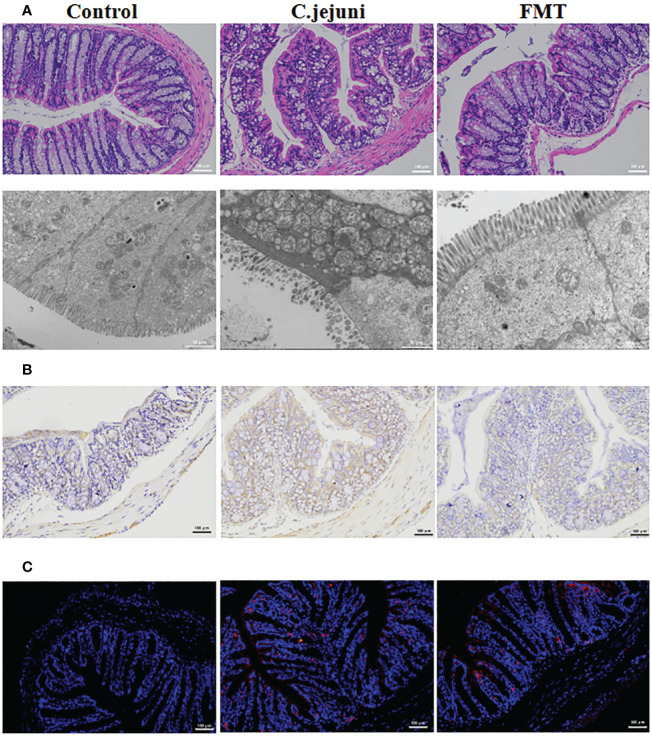
Oral deoxycholic acid alleviates *C. jejuni*-induced colitis. **(A)** Representative image of colonic tissue using H&E staining at a 200 μm field of view and transmission electron microscopy at a 2 μm field of view. **(B)** Representative image of MPO immunohistochemical staining of paraffin sections of colonic tissue (positive for brown) at 100 μm field of view. **(C)** Representative fluorescent *in situ* hybridization of selected *C. jejuni* DNA fragments from colonic tissue after representative fluorescent images at 100 μm field of view.

Bile acids are divided into primary bile acids, which are secreted by the liver and enter the digestive tract *via* the bile duct, and secondary bile acids, a group of bile acids produced by the metabolism of primary bile acids by microorganisms in the gut, which play an important role in the immunity of individuals. The addition of bile acids has previously been reported to be useful in the treatment of *C. jejuni* infection. We also wanted to investigate whether deoxycholic acid also has a therapeutic effect on *C. jejuni* infection. Freshly prepared sterile deoxycholic acid solution (10 mg/kg body weight) was administered by gavage to SPF mice infected with *C. jejuni* for 12 consecutive days to observe the therapeutic effect. The results showed that deoxycholic acid treatment alleviated the symptoms of colitis (**Figure 5A**), reduced the migration of MPO-positive neutrophils into the colonic tissue (**Figure 5B**), and reduced the infection of *C. jejuni* (**Figure 5C**). Surprisingly, however, deoxycholic acid did not have a significant effect on inflammatory factor levels (**Figure S2**).

### Proteomic data analysis

To further explore the mechanisms of *C. jejuni*-induced colitis and fecal transplant treatment, we performed proteomic analysis using tandem mass tag. Using a < 1% false discovery rate (FDR), we identified 6021 observable proteins from 21569 proteins (**Table S1A**).

**Figure 6:**
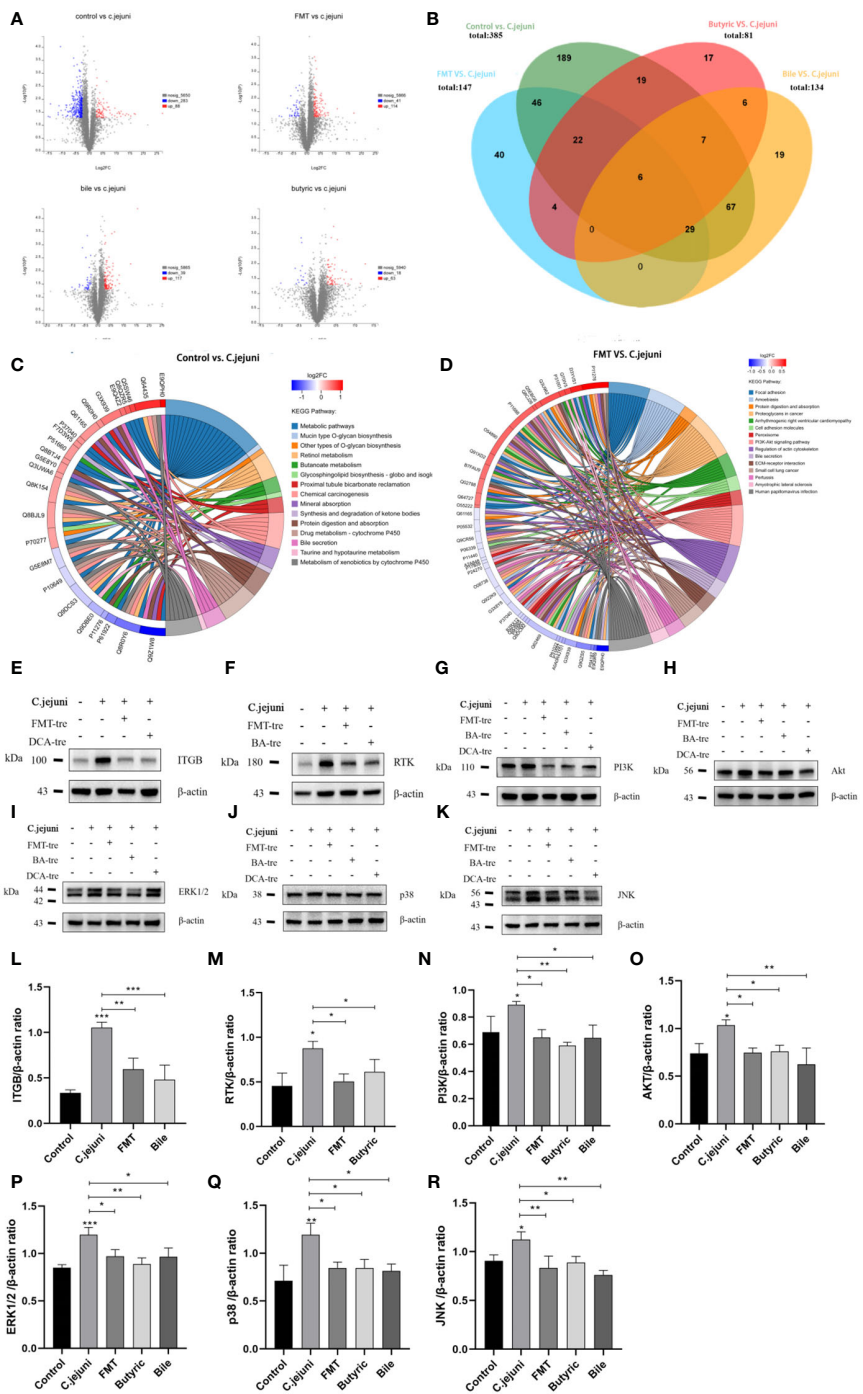
**(A)** Plots of protein volcanoes with statistically significant distributions (log p values) and magnitude of change (log2 fold change) between groups. Proteins with fold changes greater than 1.2 or less than 0.83 and p values less than 0.05 were considered to have statistically significant distributions. **(B)** Venn diagrams of protein levels between groups, reflecting the number of direct and indirect target proteins. **(C)** The chord diagram of KEGG enrichment analysis between the Control group and the *C. jejuni* group reflects the information of the KEGG signaling pathway involved in the differential proteins. **(D)** The chord diagram of KEGG enrichment analysis between the FMT group and the *C. jejuni* group reflects the information of the KEGG signaling pathway involved in the differential proteins. **(E-K)** Representative western blot brands of RTK, ITGB, PI3K, Akt, ERK1/2, JNK and p38 in the colon.ERK1/2, JNK, p38 operate on different membranes under the same conditions due to the proximity of kDA and β-actin. **(L-R)** The density analysis of RTK, ITGB, PI3K, Akt, ERK1/2, JNK and p38 western blot in the colon, n=3 for each group. Statistical significance was determined using one-way ANOVA, followed by Tukey’s test. *P ≤ 0.05, **P ≤ 0.01, ***P ≤ 0.001.

Screening at ploidy changes greater than 1.2 or less than 0.83 with p values less than 0.05, 1) infection with *C. jejuni* induced 371 differential proteins (88 upregulated and 283 downregulated) compared to the Control group; 2) compared to the *C. jejuni* group, fecal transplantation induced 155 differential proteins (114 upregulated and 41 downregulated); 3) compared to the *C. jejuni* group, butyric acid induced 81 differential proteins (63 upregulated and 18 downregulated) compared to the *C. jejuni* group; 4) deoxycholic acid induced 156 differential proteins (117 upregulated and 39 downregulated) compared to the *C. jejuni* group, and the specific differential proteins are listed (**Tables S1B-E**). The distribution of statistical significance (log p value) and magnitude of change (log2-fold change) for all proteins in each group are represented by volcano plots (**Figure 6A**). We generated Venn diagrams of protein levels between groups (**Figure 6B**) to examine the opposite correlations of changes in protein levels between *C. jejuni* infection and different treatments, with proteins or peptides in the overlap of each Venn diagram to be considered high confidence targets. We considered proteins with opposite trends of change in each treatment group compared to the *C. jejuni* group and in the *C. jejuni* group compared to the Control group as potential target proteins for the study of treatment mechanisms (e.g., A protein was up in the *C. jejuni* group/Control group comparison and down in the FMT group/*C. jejuni* group comparison, then A protein was considered a potential target protein for the FMT treatment mechanism) and FMT group, Butyric acid group, and Bile acid group with 107, 54 and 108 target proteins, respectively (**Tables S1F-H**).

### The PI3K-Akt signaling pathway and MAPK signaling pathway are core pathways for *Campylobacter jejuni* infection and therapy

Changes in the proteome occur due to specific biological pathway responses in different metabolic states - infection with *C. jejuni* and all three treatments can cause a considerable number of protein changes, i.e., the production of differential proteins. However, the metabolic and signaling pathways in which these proteins are involved are different. The significantly altered differential proteins were studied using the online managed databases GO (http://pantherdb.org) and KEGG (www.kegg.jp), which identified multiple biological pathways representing *C. jejuni* infection and resistance to *C. jejuni* associated with each therapeutic approach. We collected data from different gene databases and listed biological pathways or metabolic pathways with high significance involving at least two genes associated with the target differential protein between the groups (**Table S2**). The biological pathways or metabolic pathways involved in at least two differential protein-associated genes between the groups in the GO enrichment analysis are listed in **Tables S2A-D**, and the metabolic pathways involved in at least two differential protein-associated genes between the groups in the KEGG enrichment analysis are listed in **Supplementary Tables S2E-H**. The results of KEGG enrichment analysis between the control and *C. jejuni* groups are also shown in the form of chord diagrams in **Figure 6C**, and the results of KEGG enrichment analysis between the FMT and *C. jejuni* groups are shown in **Figure 6D**.

The PI3K-Akt pathway and MAPK pathway are widely involved in various cellular activities in animals and can be activated by inflammatory mediators to promote the production of inflammatory factors in response to inflammation, while the PI3K-Akt pathway can also activate the downstream MAPK pathway. We wondered whether such differences existed in other groups, and after researching the target proteins, we found that there were significant differences in the PI3K-Akt pathway and MAPK pathway proteins involved in all groups compared (although there were no significant differences in the pathways in the KEGG enrichment analysis). We used Western blotting to verify whether these participating proteins were significantly different. **Figures 6E-H** and **Figures 6L-O** shows WB results for four important proteins in the PI3K-Akt signaling pathway, showing that *C. jejuni* infection resulted in significantly increased levels of all proteins, activating the signaling pathway, while the opposite was true for each treatment group, inhibiting the signaling pathway. **Figures 6I-K** and **Figures 6P-R** shows the WB results of three important proteins in the MAPK signaling pathway, which has three branches related to inflammation, ERK1/2, JNK and p38, and *C. jejuni* infection also led to a significant increase in the levels of all the proteins, activating the signaling pathway, while the treatment groups inhibited it in the opposite way.

This result shows that *C. jejuni* activates the PI3K-Akt signaling pathway and MAPK signaling pathway mainly through two receptor proteins, RTK and ITGB, to infect colon tissue and induce inflammation; FMT inhibits the PI3K-Akt signaling pathway and MAPK signaling pathway by inhibiting two receptors, RTK and ITGB, whereas butyric acid only inhibits RTK to affect the PI3K-Akt signaling pathway and MAPK signaling pathway, and deoxycholic acid only inhibits ITGB to affect the PI3K-Akt signaling pathway and MAPK signaling pathway. The resistance of normal intestinal microbiota to colonization by *C. jejuni* is closely linked to the microbial metabolites butyric acid and deoxycholic acid, all of which can alleviate *C. jejuni*-induced colitis through the PI3K-Akt signaling pathway but by different mechanisms.

### *In vitro* assays validate the competitive relationship between *C. jejuni* and *Akkermansia* and the key role of the PI3K-Akt signaling pathway and MAPK signaling pathway

**Figure 7:**
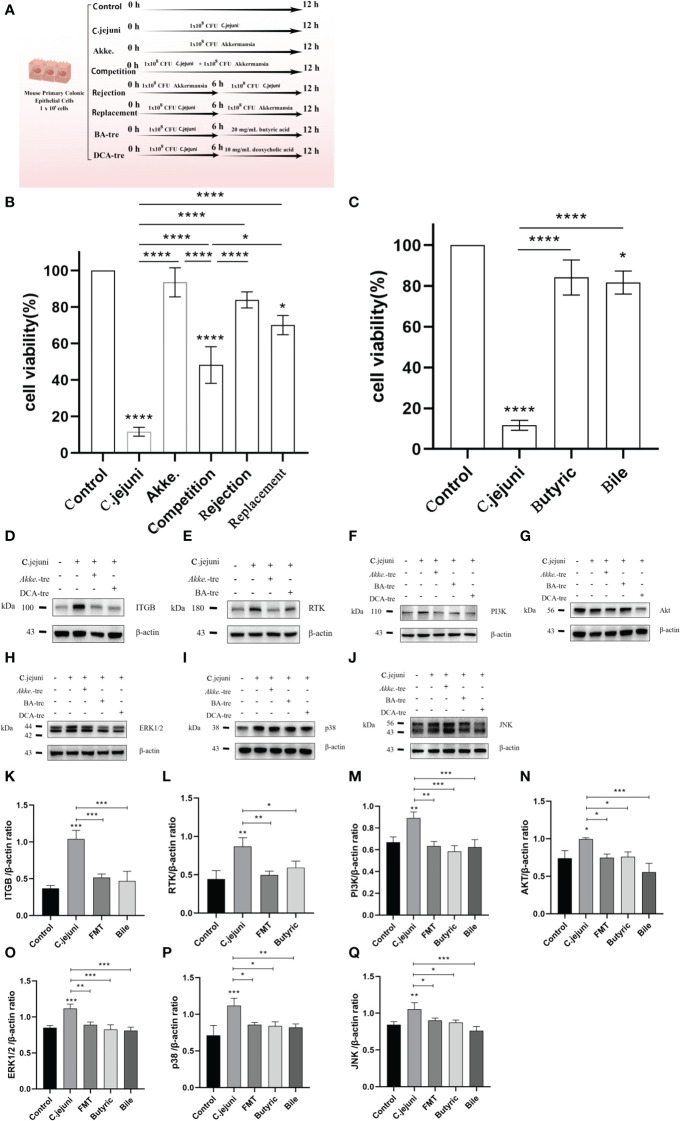
**(A)** Design diagram for *in vitro* cell experiments, n=3 for each group of parallel experiments. **(B, C)** Results of the CCK8 cell proliferation and cytotoxicity assays (n=3 for each group). **(D-J)** Representative western blot brands of RTK, ITGB, PI3K, Akt, ERK1/2, JNK and p38 in the colon.ERK1/2, JNK, p38 operate on different membranes under the same conditions due to the proximity of kDA and β-actin. **(K-Q)** The density analysis of RTK, ITGB, PI3K, Akt, ERK1/2, JNK and p38 western blot in the colon, n=3 for each group. Statistical significance was determined using one-way ANOVA, followed by Tukey’s test. *P ≤ 0.05, **P ≤ 0.01, ***P ≤ 0.001.

The cell assay design is shown in **Figure 7A** with three different invasion assay procedures (competition, rejection and replacement) and two drug treatment procedures (butyric acid and deoxycholic acid). The competition, rejection and replacement assays showed that *Akkermansia* increased the viability of *c. jejuni*-infested MUCS extremely significantly in all three methods (*P*<0.0001), but there was still a highly significant difference in cell viability in the Competition group compared to the Control group (p<0.0001), a significant difference in the Replacement group (*P*<0.05) and the highest cell viability in the Rejection group compared to the Control group. There was no significant difference (*P*>0.05). This result suggests that *Akkermansia* has both preventive and therapeutic effects on *C. jejuni* infection in the *in vitro* cell model, but the preventive effect is stronger than the therapeutic effect and that there is competition between *Akkermansia* and *C. jejuni* in the intestinal environment, and the strength of the ecological niche may be related to the timing and sequence of their colonization in the intestine (**Figure 7B**). Both butyric acid and deoxycholic acid showed good therapeutic efficacy in an *in vitro* cellular model, significantly increasing the viability of *C. jejuni*-infested MUCS (*P*<0.0001), and were still significantly different in the Deoxycholic acid group compared to the Control group (*P*<0.05)(**Figure 7C**). We also explored the expression of key proteins in the PI3K-Akt signaling pathway and MAPK signaling pathway in each group of cells. Western blot results showed that both *C. jejuni* infection increased the expression of the receptor proteins RTK and ITGB in the cells as well as the core PI3K-Akt signaling pathway. *Akkermansia* inhibited the PI3K-Akt signaling pathway and MAPK signaling pathway by inhibiting both RTK and ITGB receptors, and butyric acid only inhibited RTK affecting the PI3K-Akt signaling pathway and MAPK signaling pathway. Deoxycholic acid only inhibited ITGB to affect the PI3K-Akt signaling pathway and MAPK signaling pathway (**Figures 7D-Q**). This is consistent with the results obtained in our proteomic analysis, and thus, the *in vitro* cellular assays also validated that the PI3K-Akt signaling pathway and MAPK signaling pathway are central pathways in the infection of mouse colon tissues by *C. jejuni* and that *Akkermansia* and its metabolites butyric acid and deoxycholic acid also resist *C. jejuni* by intervening in the PI3K-Akt signaling pathway and MAPK signaling pathway.

## Discussion

**Figure 8:**
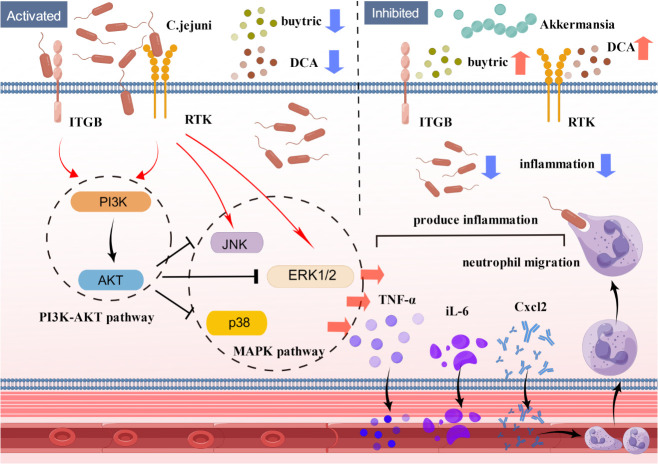
Schematic model showing the mechanism by which FMT alleviates *C. jejuni*-induced colitis. Fecal transplantation with *Akkermansia* as the core flora alleviates colitis by inhibiting the PI3K-Akt signaling pathway and MAPK signaling pathway through the bacterial metabolites butyric acid and deoxycholic acid and treating the infection of *C. jejuni* in the host organism. *C. jejuni*: *Campylobacter jejuni*, Akke.: *Akkermansia*, *DCA*: deoxycholic acid.

Our study shows that FMT can effectively treat high-dose *C. jejuni* infection. Histological and molecular analyses showed that FMT reduced inflammatory cytokine expression, neutrophil infiltration and invasion of *C. jejuni* in colonic tissue. Finally, proteomics, Western blot and *in vitro* cellular validation demonstrated that *C. jejuni* infection and treatment were closely linked to the PI3K-Akt and MAPK signaling pathways. These findings identify a novel mechanism by which Akkermansia-centered FMT inhibits the PI3K-Akt signaling pathway and MAPK signaling pathway *via* bacterial metabolites (butyric acid, deoxycholic acid) to treat *C. jejuni* infection in the host organism and alleviate colitis(**Figure 8**).

The PI3K-Akt signaling pathway and MAPK signaling pathway are core pathways in the intracellular regulatory network. Seventy-one signaling pathways are directly related to the PI3K-Akt signaling pathway, and up to 104 directly related to the MAPK signaling pathway can be identified through the KEGG database. The production of the inflammatory factors TNF-α, Cxcl2, IL-6, IL-10 and IL-22 involved in our experiments are almost all associated with these two pathways (23–26). It has been demonstrated that *C. jejuni* infection activates the PI3K-Akt signaling pathway and that the use of PI3Kγ inhibitors alleviates colitis and neutrophil infiltration (27, 28). *In vitro* experiments using *C. jejuni* to infect Caco-2 cells (a human colonic epithelial cell line) showed activation of all three branches of the MAPK pathway, ERK 1/2, JNK and p38 (29). We chose to detect PI3Kδ in WB, which is the two main subunits of PI3K expressed in immune cells, and PI3Kγ, which is more extensively studied in the immune system, compared to PI3Kδ, whose mechanism is mainly focused on the regulation of neutrophil migration in tissues (30), where the use of PI3K δ-specific inhibitors in wild-type mice treated with a model of pneumonia resulted in a significant reduction in the number of infiltrating cells in the experimental group (31). The presence of typical MPO-positive neutrophil migration in *C. jejuni*-infected colonic tissues was also reported (28, 32). This is the reason why we chose to measure PI3Kδ levels and use immunohistochemistry to detect MPO-positive neutrophil migration in colonic tissues as a basis for evaluating the severity of colitis. It has also been mentioned that *C. jejuni* promotes IL-10 expression while activating the PI3K-Akt signaling pathway and MAPK signaling pathway (27, 33), which is contrary to our results, and we believe that this may be related to the duration of the experiment, as it has been reported that IL-10 levels in the serum of patients with infectious inflammation, after an initial elevation, decline and stabilize to normal levels (34). IL-10 levels in colonic tissue were measured in our study on day 24 after infection with *Campylobacter jejuni*, which is longer than the times reported above (2 and 7 days), so we believe that a decrease in IL-10 levels in the *Campylobacter jejuni* group compared to the Control group but without significant differences is likely to occur.

*Akkermansia* is an emerging and popular research species that is widely found in the intestinal tract of animals. Current research on *Akkermansia* is mainly focused on its anticancer and obesity treatment effects, but some studies have also found that *Akkermansia* is effective in the treatment of IBD. Studies have found a significant reduction in the abundance of *Akkermansia* in the feces of IBD patients and mice with colitis, and oral administration of *Akkermansia* effectively reduced macrophage and CD8+ cytotoxic T lymphocyte (CTL) infiltration in the colon of mice with DSS-induced colitis, thereby slowing colitis (35). Some studies have confirmed that administration of *Akkermansia* improves intestinal inflammation in mice with DSS-induced ulcerative colitis (36). Some researchers suggest that a possible mechanism for *Akkermansia* to treat colitis is to increase the production of SCFAs, promote the differentiation of Treg cells and alter the composition of the intestinal flora (37). *Akkermansia* can degrade and use host mucins to produce oligosaccharides and acetate that can be used directly by butyric acid producing bacteria near the intestinal mucosa (e.g. *Anaerostipes caccae*, *Eubacterium hallii* and *Faecalibacterium prausnitzii*) (38). However, some researchers have argued against the role of *Akkermansia* in intestinal inflammation. One study proposed that *Akkermansia* aggravated the symptoms of colitis caused by *Salmonella typhimurium* infection. In the absence of *Akkermansia*, intestinal inflammation caused by a single *Salmonella typhimurium* infection was reduced with the action of IL-18 (involved in phagocytosis of intracellular pathogens), but in mice colonized by both *Akkermansia* and *Salmonella typhimurium*, levels of IL-18 were significantly reduced, thereby promoting the growth of *Salmonella typhimurium* and further exacerbating the symptoms of infection (39). Studies have also reported that gavage of *Akkermansia* led to increased severity of colitis in iL10-/- mice (40). Previous studies have shown that colitis in IL-10-/- mice is primarily caused by colonic macrophages induced by MyD88 signaling triggered by the intestinal microbiota (41). Lipopolysaccharide (LPS) from *Akkermansia* leads to higher levels of production of proinflammatory factors by colonic myeloid cells in the lamina propria, and this high LPS activity in iL10-/- mice could promote the development of colitis. Another possible explanation is that commensal bacteria in an IL-10-deficient environment instead codrive inflammation. As *Akkermansia* degrades the intestinal mucus layer, it allows more microorganisms to enter the intestinal mucosa. It has also been reported that the positive association between *Akkermansia* and intestinal inflammation is not caused by *Akkermansia* itself but occurs under specific conditions (42). In this study, the authors explored the short-term proinflammatory potential of *Akkermansia* in IL-10-/- mice and found that *Akkermansia* administration did not alter pathogenic *E. coli* NC101 infection in germ-free IL-10-/- mice; similarly, after 3 weeks of *Akkermansia* gavage, there was no indication that it promoted simplified human intestinal microbiota (SIHUMI) colonization and exacerbated inflammation in IL-10-/- mice. Therefore, gavage of *Akkermansia* did not induce or exacerbate intestinal inflammation in mice within 2-3 weeks, either in association with pathogenic *E. coli* NC101 or with SIHUMI. Taken together, these data suggest that the therapeutic effects of Akkermansia on colitis are very promising, but the mechanisms of treatment need to be further clarified (43). In contrast to the intestinal inflammation caused by substances such as DSS, the treatment of bacterial intestinal inflammation also involves complex interactions between *Akkermansia* and pathogens and between the intestinal microbiota and pathogens. The amplifying effect of some inflammatory mechanisms associated with *Akkermansia* may have exacerbated inflammation under certain specific conditions, as mentioned in the above report. This result also suggests that the study of *Akkermansia* or other probiotics in the treatment of bacterial diseases should not only discuss the properties of probiotics alone but should also be placed in the context of the overall gut microbial system, focusing on the mechanisms of microbial interactions, which may be more useful for us to investigate the mechanisms of their treatment in depth.

Our study identified the *Akkermansia*-butyric acid axis and the *Akkermansia*-deoxycholic acid axis, which play a role in the treatment of *C. jejuni* infections by preventing *C. jejuni* from invading colonic tissue and alleviating colitis. Anti-inflammatory and antibacterial effects are complementary, and many pathogens can use inflammation-related signaling pathways in the gut to enhance their own ecological niche; for example, the virulence factor *Salmonella typhimurium* induces host-driven production of a new electron receptor, even tetrasulfate, enabling it to use respiration to compete with gut microbes (44). *E. coli* uses the nitrate produced during the inflammatory response to expand its ecological niche in the intestinal microbiota (45). *C. jejuni* can also use inflammation to gain a competitive advantage over the gut microbiota for growth and thus reduce host resistance to colonization (46).

Some metabolites of the intestinal flora, such as SCFAs and bile acids, have functions related to the regulation of host receptor-mediated inflammation and immune regulation. SCFAs are signaling molecules that mediate the interaction between diet, the intestinal microbiota and the host and have important immune, metabolic and endocrine roles in the body (47). The main components, acetic acid (48), propionic acid (49) and butyric acid (50), have anti-inflammatory effects. Studies have shown that infection with *C. jejuni* also causes changes in the concentration of SCFAs in the intestinal tract (51). *In vitro* antibacterial tests have been reported on SCFAs against *C. jejuni (* 52). The current antibacterial application of SCFAs against *C. jejuni* is mainly in the disinfection of chicken products (53) or in the environmental disinfection of chicken houses (54), but there are few studies on whether SCFAs have the same therapeutic or preventive effect on the infection of *C. jejuni* in animals. Our study demonstrates that butyric acid has good therapeutic efficacy against *C. jejuni* infection in mice, and some studies have also shown that butyric acid promotes the antibacterial activity of intestinal macrophages *in vivo*, limits cell translocation *in vivo (* 55) and protects mice from *C. difficile*-induced colitis through a HIF-1-dependent mechanism (56) and that the HIF-1 signaling pathway is regulated by the PI3K-Akt signaling pathway (57). It was also found that butyrate could inhibit the motility of colorectal cancer cells *via* inactivation of the AKT/ERK signaling pathway by histone deacetylation (58), which is very close to our results that the AKT/ERK signaling pathway is also closely related to the production of inflammatory factors.

Bile acids are another group of substances closely associated with the body’s intestinal immunity. Primary bile acids are produced in the liver and transported to the intestinal lumen, where they facilitate the solubilization and absorption of dietary lipids through their detergent properties. The majority of bile acids are absorbed from the ileum and transported back to the liver *via* the bloodstream, with approximately 5% entering the large intestine, where they are modified by members of the microbiota (59). The efficacy of bile acids on immunity is broader than that of SCFAs. Two secondary bile acids have been found to increase the distribution of anti-inflammatory Treg cells and inhibit the differentiation of proinflammatory Th17 cells (60). LCA and DCA inhibit the production of proinflammatory cytokines by human peripheral blood-derived macrophages *via* the TGR5 receptor (61), and DCA produced by gut microbes inhibits the mTOR signaling pathway and alleviates *C. jejuni*-induced colitis (62). However, bile acids are not the only inhibitors of inflammation, as some studies have reported that excess bile acids stimulate FXR receptors, leading to damage to intestinal panniculocytes and exacerbating inflammation (63). Initial studies suggested that the cytotoxicity of bile acids was due to their “detergent” effect on cell membranes, such as goose deoxycholic acid (CDCA), bile acids (CA) and ursodeoxycholic acid (UDCA). Cholic acid (UDCA) causes damage to lecithinylcholine liposomes, leading to loss of hydrophobicity and membrane lysis and increased apoptosis of intestinal epithelial cells (64, 65). Increased hydrophobicity of bile acids is associated with a high rate of apoptosis in colonic epithelial cells, and coupling of bile acids with taurine or glycine appears to ameliorate this effect (66). Further evidence of the toxic effects of bile acids on intestinal mucosal barrier function in recent years can be found in relevant studies describing *in vivo* models of nonsteroidal anti-inflammatory drug (NSAID) enteropathy. Indomethacin and other NSAIDs cause linear ulcerative enteritis in rodents in a dose-dependent manner. The current understanding of this disease process includes the interaction of NSAIDs with surface phospholipids and the uncoupling of oxidative phosphorylation, which leads to an increase in intestinal mucosal permeability, exacerbated by inhibition of prostaglandin production through cyclooxygenase inhibition, with the intestinal mucosa thus exposed to bile acid attack, and the etiology of enterocolitis in this model may be due to a specific composition of bound bile acids, recirculating NSAID or a complex of microbial products (67–69). Although the NSAID enteropathy model is widely recognized by scholars, the role of bile acid attack discussed in this model remains inconclusive. Our results showed no significant changes in several inflammatory factors during deoxycholic acid treatment, possibly because we only gavaged a single dose of deoxycholic acid, which affected the balance of bile acid composition in the intestine of mice and weakened the anti-inflammatory effect on the organism. In conjunction with the results of other experiments, we believe that the addition of deoxycholic acid was generally effective in relieving *C. jejuni*-induced colitis, but this reminds us that the balance of bile acid composition in the gut also needs to be considered when treating with bile acids and that a suitable combination of bile acid preparations may be more effective than a single deoxycholic acid. Conversely, some researchers have proposed different mechanisms by which bile acids can be used to promote *C. jejuni* infection. It has been suggested that bile salts can activate CmeABC expression in *C. jejuni* and enhance resistance to multiple antimicrobial agents (70) and that the addition of 0.1% sodium deoxycholate to culture media can promote the expression of *C. jejuni* virulence-associated proteins (71). CmeABC is a member of the multidrug efflux system (commonly known as multidrug resistance pumps (MDRs)). The main role of the presence of CmeABC is to enhance the resistance of *C. jejuni* to antimicrobial compounds naturally present in the animal host (e.g., bile acid salts) to increase its ability to colonize the host gut. Excessive stimulation of CmeABC results in a significant increase in resistance of *C. jejuni* to other antibiotics (70), while on the other hand, continued overexpression of the efflux pump may also be detrimental to bacterial fitness, showing an opposite trend in resistance and colonization capacity. Some studies have shown that erythromycin-sensitive *E. coli* strains are more tolerant to bile salts (sodium deoxycholate) than erythromycin-resistant strains (72), and some studies have mentioned that continuous truncation of lipooligosaccharides (LOS), a major component of the outer membrane of *C. jejuni*, reduces bile resistance and colonization of the chicken intestine (73). After careful reading of the reports, we believe that this is not contradictory to our conclusions, as on the one hand, bile acids act by different mechanisms depending on their target, with a positive effect on the host organism in reducing intestinal inflammation and on *C. jejuni* in reducing its colonization capacity and increasing its resistance to antibiotics. The benefits of adding the right concentration and composition of bile acids may outweigh the disadvantages, but this has not been reported. This poses a new challenge for the study of bile acids in resistance to *C. jejuni*. It may be necessary to control the type and amount of bile acids and consider the issue of resistance in *C. jejuni* to avoid negative effects.

The aim of our initial trial was to investigate how to treat chronic infections of The aim of our initial trial was to investigate how to treat chronic infections with Campylobacter jejuni present in natural conditions. As mentioned in the introduction, Campylobacter jejuni is actually present in larger numbers than we thought, so there may be situations where an average human or animal is exposed to an excess of Campylobacter jejuni for a relatively long period of time and becomes infected (possibly for a few days to a few weeks). Rather than using germ-free mice (or treated WT mice with broad-spectrum antibiotics to eliminate their own gut bacteria) or immune-deficient mice as is usually the case in experiments, we conducted experiments using normal SPF-rated mice and chose to impose a longer period of infection to mimic the chronic infection situation in nature, and the duration of FMT treatment was designed to coincide with the duration of infection. The aim of our study was to investigate the mechanisms of invasion and treatment of C. jejuni under natural conditions. There may be some differences in the results compared to the elimination of the mice’s own intestinal flora, but the design of our experiments more closely resembles the natural state. Our study identified important roles for the PI3K-Akt and MAPK signaling pathways in the infection of *C. jejuni*, and these two pathways are among the most important core pathways, with extensive links to other signaling pathways in the organism. The mechanisms of these links need to be investigated in more detail. Finally, the gut microbial community is a complex ecosystem, and we believe that the therapeutic effect of FMT on *C. jejuni* is not only related to *Akkermansia*, butyric acid and deoxycholic acid but that there may be other bacteria and bacterial metabolites, either alone or in a specific combination, in the overall gut microbiota that work together with *Akkermansia* to protect the health of the host gut from *C. jejuni*, which needs to be further investigated.

Our study confirms the effectiveness of *Akkermansia*, butyric acid and deoxycholic acid in the treatment of *C. jejuni* infections. Compared to other livestock pathogens, such as *Salmonella* and *E. coli*, *C. jejuni* has been much less studied in terms of prevention and treatment mechanisms, but with the significant increase in cases of *C. jejuni* in recent years, we believe that reducing the colonization of *C. jejuni* will be one of the key directions for the medical community and livestock industry in terms of how to prevent and control Campylobacteriosis in the future. This leads to a new target direction for the application of *Akkermansia*, and there may be some kind of microbial preparation with *Akkermansia* as the core for the prevention and treatment of *C. jejuni* or other pathogens in the future, similar to the application of FMT in *C. difficile* infection. *Akkermansia* is an important component of the gut anaerobic microbial community. Our study also provides new insights into how the gut microbiota works synergistically to protect the health of the host, fight pathogen invasion, and maintain the overall stability of the gut microbial community.

## Conclusion

We have identified a novel mechanism by which FMT with *Akkermansia* as the core flora inhibits the PI3K-Akt signaling pathway and MAPK signaling pathway *via* the bacterial metabolites butyric acid and deoxycholic acid to treat *C. jejuni* infection in the host organism and alleviate colitis.

## Data availability statement

The datasets presented in this study can be found in online repositories. The names of the repository/repositories and accession number(s) can be found in the article/**Supplementary Material**.

## Ethics statement

The animal study was reviewed and approved by the Animal Protection and Utilization Committee of Zhejiang University, Zhejiang University.

## Author contributions

LJ and LQ designed the study. CY and WY collected the mice samples. WY and QH helped grow the bacteria. ZC and WW helped with cell experiments and Western blot. Bioinformatics and statistical analyses were performed by LJ and CY. WY and QH helped with data visualization. LJ wrote the manuscript. LQ revised the manuscript. All authors contributed to the article and approved the submitted version.

## Supplementary material

Supplementary Figure 1 Alpha diversity was combined considering the Shannon index **(A)**, Chao index **(B)**, and Sobs index **(C)** for the three groups. PCoA plots were assessed by PERMANOVA between the Control group and the C. jejuni group**(D)**, the C. jejuni group and the FMT group**(E)**, and the Control group and the FMT group**(F)**.The C. jejuni and FMT groups showed relative abundances of colonic bacteria at the phylum level **(G)** and genus level **(H)** in 99.5% of the communities. Analysis of differences in microbial classification between the FMT and C. jejuni groups **(I)** was shown using LEfSe analysis (LDA combined with effect size measurements).The Control group and the FMT preparation showed relative abundances of colonic bacteria at the phylum level **(J)** and genus level **(K)** in 99.5% of the communities Statistical significance was determined using one-way ANOVA, followed by Tukey’s test. * *P* ≤ 0.05, ***P* ≤ 0.01.

Supplementary Figure 2 Concentrations of TNF-α **(A)**, Cxcl2 **(B)**, IL-10 **(C)** and IL-22 **(D)** in the colonic tissue of mice in the Deoxycholic acid group (n=6). Statistical significance was determined using one-way ANOVA, followed by Tukey’s test. **P* ≤ 0.05.

Supplementary Table 1
**(A)** Statistics of protein identification information after quality control. **(B)** Statistically different proteins in the Control group compared to the *C. jejuni* group. **(C)** Statistically different proteins in the *C. jejuni* group compared to the FMT group. **(D)** Statistically different proteins in the *C. jejuni* group compared to the Butyric acid group. **(E)** Statistically different proteins in the *C. jejuni* group compared to the Deoxycholic acid group. **(F)** Potential target proteins for therapeutic mechanism studies in the FMT group. **(G)** Potential target proteins for therapeutic mechanism studies in the Butyric acid group. **(H)** Potential target proteins for therapeutic mechanism studies in the Deoxycholic acid group.

Supplementary Table 2
**(A)** Pathways with significant differences in the control vs. *C. jejuni* groups in GO enrichment analysis. **(B)** Pathways with significant differences in the *C. jejuni* vs. FMT groups in GO enrichment analysis. **(C)** Pathways with significant differences in the *C. jejuni* vs. Butyric group in GO enrichment analysis. **(D)** Pathways with significant differences in the *C. jejuni* **vs.** Bile groups in GO enrichment analysis. **(E)** Pathways with significant differences in the Control vs. *C. jejuni* groups in KEGG enrichment analysis. **(F)** Pathways with significant differences in the *C. jejuni* vs. FMT groups in KEGG enrichment analysis. **(G)** Pathways with significant differences in the *C. jejuni* vs. Butyric group in KEGG enrichment analysis. **(H)** Pathways with significant differences in the *C. jejuni* vs. Bile groups in KEGG enrichment analysis.
