# Supplementary figures and images for: Akkermansia and its metabolites play key roles in the treatment of campylobacteriosis in mice
Source: Front Immunol. 2023 Jan 12;13:1061627. doi: 10.3389/fimmu.2022.1061627 (PMC9877526; doi:10.3389/fimmu.2022.1061627)

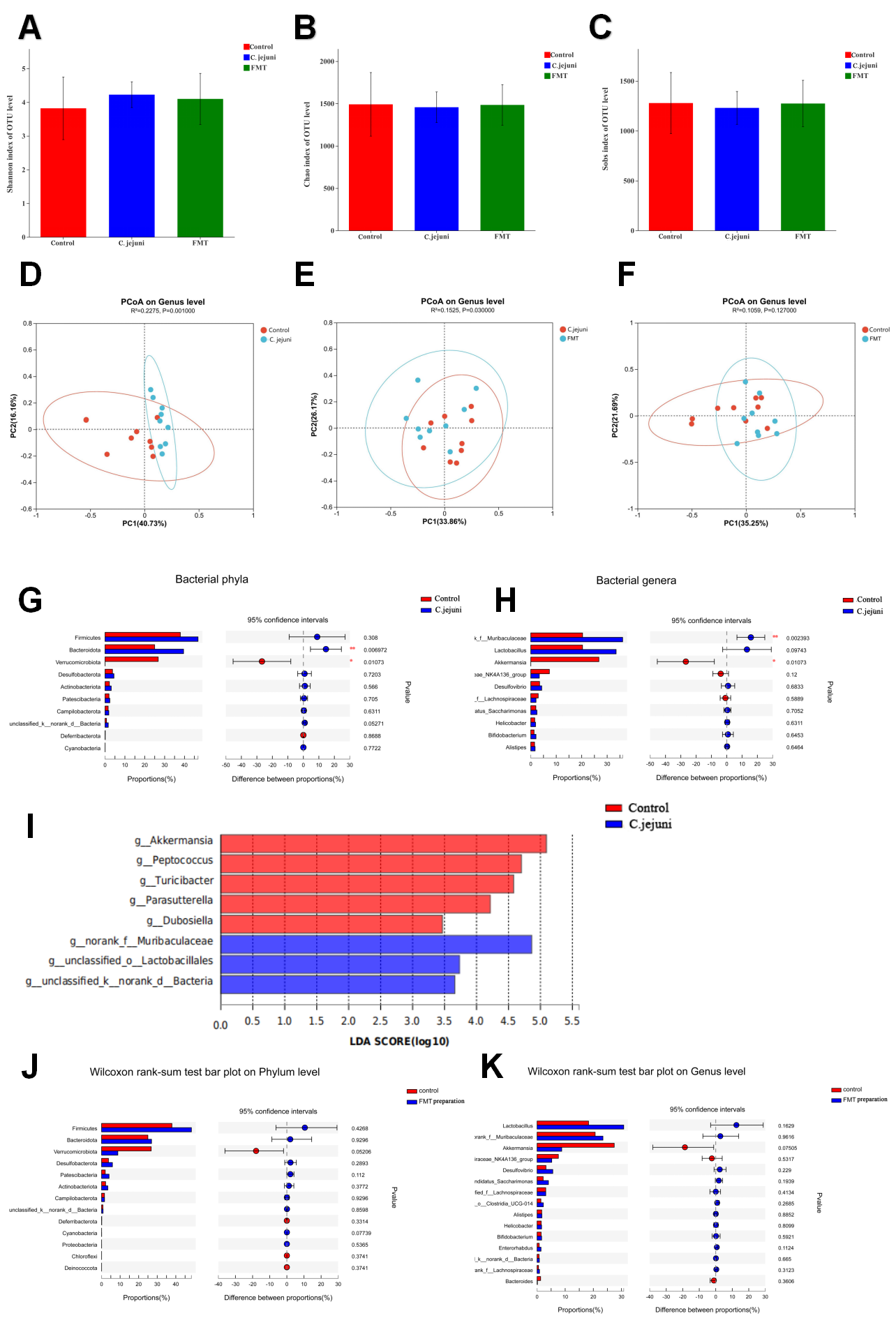

Supplement: Supplementary Figure 1 — Alpha diversity was combined considering the Shannon index (A), Chao index (B), and Sobs index (C) for the three groups. PCoA plots were assessed by PERMANOVA between the Control group and the C. jejuni group(D), the C. jejuni group and the FMT group(E), and the Control group and the FMT group(F).The C. jejuni and FMT groups showed relative abundances of colonic bacteria at the phylum level (G) and genus level (H) in 99.5% of the communities. Analysis of differences in microbial classification between the FMT and C. jejuni groups (I) was shown using LEfSe analysis (LDA combined with effect size measurements).The Control group and the FMT preparation showed relative abundances of colonic bacteria at the phylum level (J) and genus level (K) in 99.5% of the communities Statistical significance was determined using one-way ANOVA, followed by Tukey’s test. * P ≤ 0.05, **P ≤ 0.01. [file DataSheet_1.zip › Figure S1.tif]

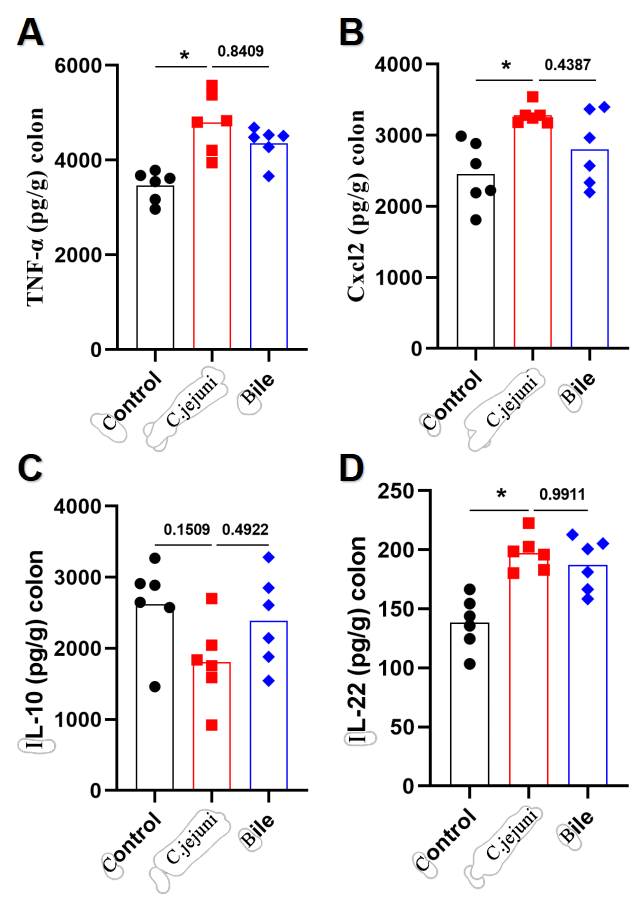

Supplement: Supplementary Figure 1 — Alpha diversity was combined considering the Shannon index (A), Chao index (B), and Sobs index (C) for the three groups. PCoA plots were assessed by PERMANOVA between the Control group and the C. jejuni group(D), the C. jejuni group and the FMT group(E), and the Control group and the FMT group(F).The C. jejuni and FMT groups showed relative abundances of colonic bacteria at the phylum level (G) and genus level (H) in 99.5% of the communities. Analysis of differences in microbial classification between the FMT and C. jejuni groups (I) was shown using LEfSe analysis (LDA combined with effect size measurements).The Control group and the FMT preparation showed relative abundances of colonic bacteria at the phylum level (J) and genus level (K) in 99.5% of the communities Statistical significance was determined using one-way ANOVA, followed by Tukey’s test. * P ≤ 0.05, **P ≤ 0.01. [file DataSheet_1.zip › Figure S2.tif]
